# What is the cause of this patient's persistent fever?

**DOI:** 10.1097/01.JAA.0000832652.64634.66

**Published:** 2022-07-26

**Authors:** Amy Kole

**Affiliations:** **Amy Kole** practices in the division of infectious diseases at Mayo Clinic in Phoenix, Ariz. The author has disclosed no potential conflicts of interest, financial or otherwise.

## CASE

A 35-year-old man was referred to the infectious diseases clinic because of persistent fevers for 3 weeks and an elevated d-dimer.

### History

About 3 weeks ago, the patient was diagnosed with COVID-19 by his primary care provider (PCP) following a positive nasopharyngeal SARS-CoV-2 RNA polymerase chain reaction test. He continued to have intermittent fevers at home up to 102° F (38.9° C). Associated symptoms included shortness of breath, persistent fatigue, and diffuse myalgia.

One week ago, the patient returned to his PCP for further evaluation. Because of concern for a pulmonary embolism (PE), a d-dimer was obtained and was 844 ng/mL (normal range, less than 500 ng/mL). His PCP subsequently referred the patient to the ED.

In the ED, a complete blood cell count with differential was significant for a leukocytosis of 13,500 cells/mm^3^ with a neutrophilic predominance. A comprehensive metabolic panel was unremarkable with all values within normal limits. A CT angiogram of the chest was performed and excluded PE.

Chest imaging revealed diffuse centrilobular nodularity throughout lungs bilaterally with multiple nodules demonstrating adjacent ground-glass opacity. Additionally, prominent hilar and mediastinal adenopathy were noted. The patient was deemed medically stable for discharge and told to follow up in the outpatient infectious diseases clinic.

## DIFFERENTIAL DIAGNOSIS

- persistent COVID-19
- community-acquired pneumonia
- fungal pneumonia

## OUTCOME

In the infectious diseases clinic, the patient was in no acute distress and was alert and oriented to person, place, and time. Vital signs were BP, 142/85 mm Hg; pulse, 102; respirations, 18; oral temperature, 36.5° C (97.7° F); and SpO_2_, 96% on room air. His lungs were clear to auscultation bilaterally. He had a regular cardiac rate and rhythm without murmurs, rubs, or gallops. His skin was warm and dry with no rashes. An abdominal examination was benign with normoactive bowel sounds and no tenderness to palpation. The musculoskeletal examination also was unremarkable, with normal range of motion and no deformities or edema.

He did not endorse coughing at any point throughout his illness. He also denied any abdominal pain, nausea, vomiting, or diarrhea. Review of systems was negative for headaches, rashes, or night sweats. The patient was born and raised in Phoenix, Ariz., and still lives there. He denied any recent travel or sick contacts. The patient works in finance. He worked from home for the past 6 months since the start of the COVID-19 pandemic. He is a lifelong nonsmoker and denied illicit drug or tobacco use. He denied any significant past medical history. He did not take any medications or supplements.

The patient's image findings from the ED were incompatible with a typical community-acquired pneumonia because he did not have a focal consolidation. The findings also were not consistent with COVID-19, which generally demonstrates diffuse ground-glass opacities rather than nodular disease.

Endemic fungal pneumonia was suspected, and *Coccidioides* serologies were ordered. *Coccidioides* IgM and IgG were positive by enzyme immunoassay (EIA). Additionally, *Coccidioides* IgG was positive by immunodiffusion with a complement fixation titer of 1:32. The patient was diagnosed with pulmonary coccidioidomycosis and was started on antifungal therapy. He completed a 3-month course of oral fluconazole 400 mg daily. Following treatment, his symptoms gradually resolved, and his pulmonary nodules decreased in size.

## DISCUSSION

Coccidioidomycosis, known colloquially as valley fever, is a fungal infection endemic to the southwestern United States. The disease is caused by inhalation of the arthroconidium of *Coccidioides immitis* and *Coccidioides posadasii*.^1^ *Coccidioides* spores are not easily aerosolized and thus not transmissible from person to person.^1^ Incubation time from exposure to symptom onset can range from 7 to 28 days.^2^

Clinical presentation and severity of pulmonary coccidioidomycosis are variable. Most patients present with one or more of the following symptoms: fevers, night sweats, cough, shortness of breath, myalgia, arthralgia, rashes, weight loss, and poor appetite.^1^ Common radiographic findings include a lobar or segmental pneumonia with hilar adenopathy, though a multifocal pneumonia is seen in some patients.^2^ As the infection resolves, a residual pulmonary nodule or small cavity often remains.^2^

The diagnosis is primarily established by serum antibody detection via enzyme immunoassay as well as immunodiffusion and complement fixation.^3^ Alternatively, the diagnosis can be confirmed by isolating *Coccidioides sp*. on cultures obtained from a biopsy or bronchoscopy with alveolar lavage.^4^ This approach is particularly advantageous in immunocompromised patients who may have negative coccidioidal serologies despite an active infection.^5^

Treatment varies according to the host and severity of disease. Guidelines from the Infectious Diseases Society of America recommend close observation and supportive care for immunocompetent patients with mild or resolving symptoms.^4^ Antifungal therapy is recommended for patients with significant debilitating disease, extensive pulmonary involvement, or those with significant comorbidities.^4^ First-line antifungal treatment is oral fluconazole 400 mg daily which has high bioavailability and good tolerability.^1^ Duration of treatment is not established, but experts recommend between 3 to 6 months depending on clinical response.^4^ In most cases, symptoms fully resolve and do not require prolonged antifungal therapy.

In about 1% of immunocompetent patients with coccidioidomycosis, *Coccidioides* disseminates to extrapulmonary sites such as the skin, central nervous system, or musculoskeletal system, although dissemination to any organ is possible.^2^ Men, patients with diabetes, and patients of African or Filipino descent are at a higher risk of dissemination and severe disease.^2^

Many experts would view any of these characteristics as an indication for antifungal treatment.^4^ Additionally, patients with impaired cellular immunity, such as those with HIV, solid organ transplant, hematopoietic stem cell transplant, or those taking a biologic agent, are at an increased risk of severe infection, disseminated infection, and death.^5^,^6^ These patients often require prolonged or even lifelong antifungal treatment.^5^

Coccidioidomycosis acquired during pregnancy poses challenges. Pregnancy is a known risk factor for disseminated coccidioidomycosis.^7^ Expert opinion on the management of coccidioidomycosis during pregnancy varies. Azole antifungals can be teratogenic and should be avoided during the first trimester. Data are limited about the safety of azoles in the second and third trimesters, though some studies suggest they may be safe later in pregnancy.^7^ Therefore, some experts recommend empiric treatment with IV liposomal amphotericin B, particularly during the first trimester.^4^ Other experts recommend no empiric treatment if the patient has frequent clinic follow-up and *Coccidioides* serologies monitored every 6 to 12 weeks.^7^

## CONCLUSION

Coccidioidomycosis is a common cause of pneumonia in the southwestern United States. The diagnosis should be considered in patients presenting with pneumonia who have recent travel or residence in this area. Clinical presentation varies, but most patients present with nonspecific symptoms including cough, fevers, night sweats, shortness of breath, and arthralgia. Antifungal therapy is recommended for patients with moderate to severe disease, immunocompromising conditions, or risk factors for dissemination.
